# 1-Oleyl-lysophosphatidic acid (LPA) promotes polarization of BV-2 and primary murine microglia towards an M1-like phenotype

**DOI:** 10.1186/s12974-016-0701-9

**Published:** 2016-08-26

**Authors:** Ioanna Plastira, Eva Bernhart, Madeleine Goeritzer, Helga Reicher, Vishwanath Bhat Kumble, Nora Kogelnik, Andrea Wintersperger, Astrid Hammer, Stefanie Schlager, Katharina Jandl, Akos Heinemann, Dagmar Kratky, Ernst Malle, Wolfgang Sattler

**Affiliations:** 1 Institute of Molecular Biology and Biochemistry, Medical University of Graz, Harrachgasse 21, 8010 Graz, Austria; 2 BioTechMed-Graz, Graz, Austria; 3 Institute of Cell Biology, Histology and Embryology, Medical University of Graz, Graz, Austria; 4 Institute of Experimental and Clinical Pharmacology, Medical University of Graz, Graz, Austria

**Keywords:** IL-4, IL-10, LPS, M1 and M2 polarization, Neuroinflammation

## Abstract

**Background:**

Microglia, the immunocompetent cells of the CNS, rapidly respond to brain injury and disease by altering their morphology and phenotype to adopt an activated state. Microglia can exist broadly between two different states, namely the classical (M1) and the alternative (M2) phenotype. The first is characterized by the production of pro-inflammatory cytokines/chemokines and reactive oxygen and/or nitrogen species. In contrast, alternatively activated microglia are typified by an anti-inflammatory phenotype supporting wound healing and debris clearance. The objective of the present study was to determine the outcome of lysophosphatidic acid (LPA)-mediated signaling events on microglia polarization.

**Methods:**

LPA receptor expression and cyto-/chemokine mRNA levels in BV-2 and primary murine microglia (PMM) were determined by qPCR. M1/M2 marker expression was analyzed by Western blotting, immunofluorescence microscopy, or flow cytometry. Cyto-/chemokine secretion was quantitated by ELISA.

**Results:**

BV-2 cells express LPA receptor 2 (LPA2), 3, 5, and 6, whereas PMM express LPA1, 2, 4, 5, and 6. We show that LPA treatment of BV-2 and PMM leads to a shift towards a pro-inflammatory M1-like phenotype. LPA treatment increased CD40 and CD86 (M1 markers) and reduced CD206 (M2 marker) expression. LPA increased inducible nitric oxide synthase (iNOS) and COX-2 levels (both M1), while the M2 marker Arginase-1 was suppressed in BV-2 cells. Immunofluorescence studies (iNOS, COX-2, Arginase-1, and RELMα) extended these findings to PMM. Upregulation of M1 markers in BV-2 and PMM was accompanied by increased cyto-/chemokine transcription and secretion (IL-1β, TNFα, IL-6, CCL5, and CXCL2). The pharmacological LPA5 antagonist TCLPA5 blunted most of these pro-inflammatory responses.

**Conclusions:**

LPA drives BV-2 and PMM towards a pro-inflammatory M1-like phenotype. Suppression by TCLPA5 indicates that the LPA/LPA5 signaling axis could represent a potential pharmacological target to interfere with microglia polarization in disease.

## Background

Microglia are the resident immune cells of the brain and are endowed with specific receptor sets that are able to detect subtle alterations of the finely tuned micromilieu in the central nervous system (CNS) [1, 2]. Even in the resting state, the dynamic microglia processes scan the CNS environment and respond to danger signals [3]. Neuronal injury results in the release of ATP, neurotransmitters, growth factors and cytokines, or in changes of local ion homeostasis resulting in microglia activation [4]. Depending on the signal encountered, microglia can activate different repair programs that determine the severity of the response [4]. These responses include morphological transformation (increased size of cell bodies, thickening of proximal processes, decreased ramification of distal branches), proliferation, migration, phagocytosis, and the production of bioactive molecules [5]. These events are presumably the first steps that mobilize the cellular and molecular defense machinery in the CNS leading to subsequent microglia activation. Depending on the environmental milieu and stimulus encountered, this activation profile of microglia can reach from classically activated (M1) to alternatively activated (M2) cells [6].

Lysophosphatidic acid (LPA) is a mixture of saturated or unsaturated acyl/alkyl residues at the sn-1 or sn-2 position [7] that are present in biological fluids including cerebrospinal fluid (CSF) [8]. LPA is produced through different pathways by means of phospholipase A_1_- and A_2_-mediated hydrolysis of phosphatidic acid [8], de novo synthesis from glycerol-3-phosphate via acyltransferases [9], or autotaxin (ATX)-dependent cleavage of lysophosphatidylcholine [10]. The majority of circulating LPA is thought to originate from ATX [11]. In line, LPA levels in mice heterozygous for the ATX allele are reduced by 50 % [12–14]. Tissue distribution analyses revealed that ATX is expressed in murine and human brain [15]. Consistently, the brain contains significant levels of LPA [16], which increase in response to CNS injury [17–19]. In vitro and in vivo data demonstrated that LPA receptor-mediated signaling cascades play prominent roles in the CNS [20, 21].

The effects of LPA are mediated through the six currently recognized G protein-coupled receptors termed LPA receptors 1-6 (LPA1-6), which couple to one or more heterotrimeric G proteins [21, 22]. All members of the LPA receptor family are expressed in the CNS and regulate blood-brain barrier permeability [23], neuroprogenitor cell function [24], synaptic transmission [25], myelination [26], and brain immune responses [27–29]. Microglia express a range of LPA receptors that regulate cell morphology [30], membrane ruffling and hyperpolarization [31], metabolic changes [27], migration and ion mobilization [29], and growth factor production [31]. Recently, it was demonstrated that activation of LPA1 contributes to demyelination in spinal cord injury and that these effects are partly mediated by activated microglia [19].

In the peripheral immune system, LPA may adopt the role of sphingosine-1-phosphate during lymphocyte egress [32]. ATX is expressed in lymphoid organ high endothelial venules, where it generates LPA and promotes the entry of lymphocytes into lymphoid organs [33, 34]. It was suggested that local synthesis of LPA by ATX provides a chemotactic signal that facilitates T cell exit from the circulation into the lymph nodes [35]. In accordance, 1-bromo-3(S)-hydroxy-4-(palmitoyloxy)butyl]phosphonate (BrP-LPA), which antagonizes ATX and LPA receptor function, attenuated trafficking of lymphocytes into lymph nodes [36]. LPA potently affects migration, chemotaxis, proliferation, survival signaling, and interleukin-1β (IL-1β) secretion in different cell types of the immune system (reviewed in [35]). In monocyte-derived dendritic cells, LPA upregulates expression of CD86 (M1 marker), thereby enhancing T cell proliferation [37].

Unlike other CNS resident cells, microglia originate from hematopoietic stem cells in the yolk sac and act as primary cells responding to pathogens or injuries of the CNS. Similarly to macrophages, microglia polarize to an M1 phenotype (e.g. in response to lipopolysaccharide; LPS) in order to produce pro-inflammatory mediators [38]. In contrast, the anti-inflammatory cytokine IL-4 gives rise to the formation of a protective M2 microglia population in a murine stroke model [39]. Based on this evidence, it is conceivable that mediators controlling M1/M2 polarization of microglia could impact on promotion or resolution of neuroinflammation in the CNS.

In earlier studies, we showed that LPA increases the expression of several glycolytic enzymes in microglia [27], indicating a shift towards a pro-inflammatory M1 polarization [40]. We suggest that LPA may act as a potent regulator of microglia signaling, thereby modulating microglia polarization.

## Methods

### Materials

Cell culture medium RPMI1640 and DMEM (Dulbecco’s modified Eagle’s medium), fetal calf serum (FCS), antibiotics, and 0.25 % trypsin were from Invitrogen (Waltham, MA, USA). LPA (1-oleoyl-2-hydroxy-sn-glycero-3-phosphate) was from Sigma-Aldrich (St. Louis, MO, USA). Recombinant murine IL-4 and IL-10 were from Peprotech (Rocky Hill, NJ, USA) and LPS from *E. coli* (O111:B4) was from Sigma-Aldrich (St. Louis, MO, USA). Antibodies against cyclooxygenase 2 (COX-2) and arginase-1 (Arg-1; used only for Western blotting) were purchased from Cell Signaling (Beverly, MA, USA), inducible nitric oxide synthase (iNOS) antibody was from BD Biosciences (San Jose, CA, USA). For immunofluorescence, the COX-2 and Arg-1 antibodies were from Santa Cruz (Dallas, TX, USA), the antibodies against resistin-like alpha (RELMα, alternative name FIZZ-1) and iNOS were from Abcam (Cambridge, UK) and the CD11b antibody was from Novus Biologicals (Littleton, CO, USA). β-Tubulin monoclonal antibody, β-actin and tomato-lectin were from Sigma-Aldrich (St. Louis, MO, USA). Alexa Fluor® 488 Phalloidin was from Invitrogen (Waltham, MA, USA). Phycoerythrin (PE)-CD40, allophycocyanin (APC)-CD86, PE-CD206 antibodies and their isotype controls were from e-Bioscience (San Diego, CA, USA). 5-(3-Chloro-4-cyclohexylphenyl)-1-(3-methoxyphenyl)-1H-pyrazole-3-carboxylic acid (TCLPA5) was from Tocris Bioscience (Bristol, UK) and BrP-LPA was from Echelon Biosciences (Salt Lake City, UT, USA). *tert*-Butyl hydroperoxide was from Sigma-Aldrich. ELISA development kits were from Peprotech (Rocky Hill, NJ, USA). Primers were from QIAGEN or Invitrogen. Kits that were used for quantitative real-time PCR (qPCR) analysis were purchased from QIAGEN (Hilden, Germany) or Applied Biosystems (Foster City, CA, USA).

### BV-2 microglia

The BV-2 murine microglial cell line was purchased from Banca Biologica e Cell Factory (Genova, Italy). Cells were grown and maintained in RPMI1640 medium supplemented with 10 % FCS, 1 % penicillin/streptomycin, and 1 % glutamine and cultured under standard conditions. Culture medium was changed to fresh medium every 2 or 3 days and when the cells reached confluence, they were splitted into new flasks or used immediately for the experiments.

### Primary microglia isolation and culture

Primary murine microglia (PMM) were isolated and purified from cortices of neonatal (P0–P4) mice as previously described [41]. In brief, cortices were isolated from the whole brain, stripped from their meninges, and minced into small pieces. Glial cells were separated by trypsinization (0.1 % trypsin, 20 min, 37 °C, 5 % CO_2_), and the cell suspension was cultured in 75 cm^2^ tissue culture flasks precoated with 5 mg/ml poly-d-lysine in DMEM containing 15 % FCS, 1 % penicillin/streptomycin, and 1 % glutamine. After 2 to 3 days in culture, the medium was changed to DMEM/10 % FCS and cells were cultured for additional 10 to 14 days. Microglia were removed from the astrocytic monolayer by smacking the flasks 10-20 times and seeded onto poly-d-lysine-coated cell culture plates for further use. The purity of primary murine microglia was determined by immunocytochemistry with anti-CD11b or tomato-lectin staining and was always ≥95 %.

### qPCR analysis

Total RNA was extracted from BV-2 or primary microglia cells using the RNeasy Mini or RNeasy Micro kit (QIAGEN, Hilden, Germany) and quantified using NanoDrop (Thermo Fisher Scientific, Waltham, MA, USA). RNA was reverse-transcribed by using the high-capacity cDNA reverse transcription kit (Applied Biosystems, Foster City, CA, USA) or by using the SuperScript® III reverse transcription kit (Invitrogen, Waltham, MA, USA). Quantitative real-time PCR (qPCR) was performed on an Applied Biosystems 7900HT Fast Real-Time PCR System using the QuantifastTM SYBR® Green PCR kit (QIAGEN, Hilden, Germany). Amplification of murine hypoxanthine-guanine phosphoribosyltransferase (HPRT) as housekeeping gene was performed on all samples as internal controls to account for variations in mRNA levels. Expression profiles and associated statistical parameters were analyzed by the 2^-ddCt^ method [42]. Gene specific primers were purchased from QIAGEN (LPA receptors) and Invitrogen (cytokines and chemokines). Primer sequences are listed in Tables 1 and 2. In case of non-detects, we discriminate between undetermined values (that do not exceed the Ct threshold) and absent values (no reaction occurred) [43].

**Table 1:** Primers (QIAGEN) used for qPCR analyses of LPA receptor expression

Gene	Cat. no.	Product length (bp)
Hprt	QT00166768	168
Lpar1	QT00107709	94
Lpar2	QT00106008	94
Lpar3	QT00264320	99
Lpar4	QT00125888	96
Lpar5	QT00312571	100
Lpar6	QT00325668	118

**Table 2:** Primers (Invitrogen) used for expression analyses of cytokines and chemokines by qPCR

Gene	Forward primer (5′–3′)	Reverse primer (5′–3′)	Product length (bp)
Ccl5	GCTGCTTTGCCTACCTCTCC	TCGAGTGACAAACACGACTGC	104
Cxcl2	AGTGAACTGCGCTGTCAATG	GCCCTTGAGAGTGGCTATGA	126
Il1β	TGTGAAATGCCACCTTTTGA	GGTCAAAGGTTTGGAAGCAG	94
Il6	TGATGCACTTGCAGAAAACA	ACCAGAGGAAATTTTCAATAGGC	109
Tnfα	CCACCACGCTCTTCTGTCTAC	AGGGTCTGGGCCATAGAACT	103

### LPA treatment

For LPA treatment, BV-2 and PMM were plated in poly-d-lysine precoated plates (different sizes according to each experiment) and allowed to adhere for 2–3 days. Before treatments, cells were always incubated in serum-free DMEM overnight (o/n). The following day, medium was changed to serum-free DMEM containing 0.1 % BSA (control) or DMEM containing 0.1 % BSA and LPA (1 μM). BSA was used as LPA carrier. Aqueous LPA stock solutions (10 mM) were stored at −70 °C. Only freshly thawed stock solutions were used for the experiments. LPA solutions, culture medium, BSA, and PBS that were used during the experiments were tested for endotoxin content using the Limulus amebocyte lysate test. Endotoxin content was always <0.5 EU/ml.

### Pharmacological inhibition of LPA receptors

We used BrP-LPA, a pan LPA receptor/ATX antagonist [44] and TCLPA5, a specific antagonist for LPA5 [45]. BrP-LPA was diluted in distilled water (stock concentration of 2 mM), aliquoted and kept at −20 °C. TCLPA5 was diluted in dimethylsulfoxide (DMSO) (stock concentration 100 mM) and kept at −20 °C. TCLPA5 solutions are stated to be stable for maximum 40 days. During the experiments, both antagonists were used at a final concentration of 5 μM. Cells were pretreated with the antagonists for 2 h before starting the experiment.

### Immunoblotting

For Western blotting experiments, BV-2 cells were seeded onto 6-well plates at a density of 1 × 10^5^ cells/well. Prior to experiments, cells were cultured in serum-free medium (o/n) and then incubated in serum-free medium containing 0.1 % BSA in the presence of 1 μM LPA or 1 μM LPA in the absence or presence of BrP-LPA (5 μM) or TCLPA5 (5 μM) for the indicated time periods. After removing the supernatant, cells were washed twice with ice-cold PBS and lysed in RIPA buffer (50 mM Tris-HCl pH 7.4, 1 % NP-40, 150 mM NaCl, 1 mM Na_3_VO_4_, 1 mM NaF, 1 mM EDTA) containing protease inhibitors (aprotinin, leupeptin, pepstatin: 1 μg/ml each), 10 μM PMSF, and phosphatase inhibitors (Thermo Scientific, Vienna, Austria). Cells were mechanically scraped off using a rubber scraper and centrifuged at 13,000 rpm for 10 min. Protein concentrations were determined using the BCA kit (Thermo Scientific) using BSA as a standard. One hundred micrograms of total cell protein was loaded per lane and separated by SDS-PAGE (10 %). After electrophoresis, proteins were transferred to polyvinylidene difluoride membranes using electrophoretic transfer (Bio-Rad, Berkeley, CA, USA). Membranes were blocked with 5 % low-fat milk in TBST for 2 h at room temperature and incubated with the following primary antibodies: anti-iNOS (1:500), anti-Arg-1 (1:1000), and anti-COX-2 (1:1000). After removal of primary antibodies, the membranes were washed for 30 min in TBST and incubated for 2 h at room temperature with HRP-conjugated secondary antibodies (anti-rabbit 1:10,000; anti-mouse 1:5000). After washing with TBST for 1 h, immunoreactive bands were visualized using ECL or ECL plus reagents (Thermo Scientific) and detected with a chemiluminescence detection system (ChemiDoc Bio-Rad, Berkeley, CA, USA).

### Immunofluorescence

To examine changes in cell morphology in response to LPA treatment, BV-2 and primary microglia were stained for β-tubulin or F-actin. After seeding, cells were serum-starved (untreated) or incubated in the presence of 1 μM LPA (24 h). Then, cells were washed with pre-warmed PBS, fixed with 4 % paraformaldehyde/PBS for 10 min, and permeabilized with 0.5 % TritonX/PBS for 10 min at room temperature. Following washing with PBS, cells were incubated with blocking buffer (Thermo Scientific, Waltham, MA, USA) for 1 h at 4 °C. Incubations with anti-β-tubulin (1:100) or phalloidin (1:50) were carried out overnight at 4 °C. Finally, cells stained for β-tubulin were incubated with cyanine (Cy)-3-labeled secondary antibody (1:200, 30 min, room temperature). Microglia nuclei were counterstained with Hoechst 33342 (Invitrogen, Waltham, MA, USA). All slides were washed three times with PBS and mounted using a mounting medium (Dako Austria GmbH, Vienna, Austria).

To identify changes in M1 and M2 markers, BV-2 and PMM were seeded onto poly-d-lysine-coated chamber slides at a density of 4 × 10^4^ and 1 × 10^5^ cells, respectively, and incubated in serum-free medium in the absence or presence of LPA (1 μM) or LPA plus TCLPA5 (5 μM). LPS (20 ng/ml) and IL-4 (40 ng/ml) were used as positive controls for M1 and M2 polarization of PMM. Fixation, permeabilization, and blocking steps were performed as described above. Following blocking of unspecific binding, cells were incubated with FITC-conjugated tomato lectin (1:100) and antibodies against iNOS (1:20), Arg-1 (1:50), COX-2 (1:20), or RELMα (1:50), followed by incubation with labeled secondary antibodies. Slides were counterstained with Hoechst 33342. Confocal fluorescence microscopy imaging was performed using a Leitz/Leica TCSSP2 microscope (Leica Lasertechnik GmbH, Heidelberg, Germany). Quantification of fluorescence intensity was performed using ImageJ. At least 50 cells out of 3 different areas per chamber were measured.

### Flow cytometry

Flow cytometry was used in order to assess the expression of CD40, CD86, and CD206 in microglia cells. BV-2 and PMM were seeded in triplicate onto 6-well and poly-d-lysine coated 24-well plates at a density of 1 × 10^5^ and 1.5 × 10^5^ cells per well, respectively. After 24 h serum starvation, cells were incubated in the presence of 1 μM LPA for 12, 24, and 48 h. BV-2 cells were also used to test effects of the inhibitors on surface marker expression. Serum-starved cells were incubated with vehicle controls, LPA, or LPA plus the antagonists for the abovementioned time periods. Cells were then collected, blocked using the Ultra V blocker (Thermo Scientific), and incubated with PE anti-CD40, APC anti-CD86, or PE anti-CD206 antibody (1:50). Finally, cells were fixed and measured using a Guava easyCyte 8 Millipore flow cytometer.

### ELISA

IL-1β, tumor necrosis factor α (TNFα), interleukin-6 (IL-6), CCL5 (RANTES), and C-X-C motif chemokine 2 (CXCL2, alternative name MIP-2) concentrations in the cellular supernatant were quantitated using murine ELISA development kits (Peprotech, NJ, USA). Briefly, BV-2 and PMM were seeded in triplicate onto 12-well and poly-d-lysine coated 24-well plates at a density of 1 × 10^5^ and 2.5 × 10^5^ cells per well, respectively. After serum starvation (o/n), cells were incubated in serum-free medium, containing LPA in the absence or presence of BrP-LPA or TCLPA5 for the indicated times. For each time point, the supernatants were collected and kept at −70 °C until further use. The assays were performed according to manufacturer’s instructions. The standard curve for each ELISA was performed in triplicate. The concentrations of the cytokines and chemokines were determined using the external standard curve.

### NO detection

iNOS activity was assessed indirectly by measuring the accumulated total nitrate levels in the supernatant after 12, 24, and 48 h in the case of BV-2 cells and 2, 8, 24, and 48 h in the case of primary microglia cells, using the total nitric oxide assay kit (ENZO Life Sciences, Switzerland). In this Griess assay, nitrate is reduced to nitrite by means of nitrate reductase. Fifty microliters of supernatant from each sample were processed according to manufacturer’s protocol. A standard curve was generated in the range between 0 and 100 μM using nitrate as standard. The total nitrate concentration per sample was determined using the external calibration curve.

### Measurement of carboxy-H_2_DCFDA oxidation

Intracellular reactive oxygen species (ROS) levels were measured using the 2′,7′-dichlorofluorescin diacetate (DCFDA) cellular ROS detection kit (Abcam, Cambridge, UK). After internalization and subsequent hydrolysis, the redox indicator probe carboxy-H_2_DCFDA is converted to carboxy-H_2_DCF, which in the presence of oxidant species is oxidized to fluorescent carboxy-DCF [46]. BV-2 and PMM were seeded in black clear bottom 96-well plates at a density of 2.5 × 10^4^ cells per well. Cells were allowed to adhere overnight and then incubated with 20 μM DCFDA for 40 min at 37 °C in the dark. The solution was removed and the cells were treated with vehicle control (DMSO), LPA, or LPA plus TCLPA5 for 0.5, 1, 3, and 6 h. Fluorescence intensity was measured with excitation and emission wavelengths of 485 and 535 nm, respectively.

### Statistical analysis

All experiments were performed in triplicate and repeated at least three times. Data are presented as mean + SD unless otherwise stated. Statistical significance was determined by one-way ANOVA with Bonferroni correction using GraphPad 5.0 Prism. Values of *p* ≤ 0.05 were considered significantly different.

## Results

**Fig. 1:**
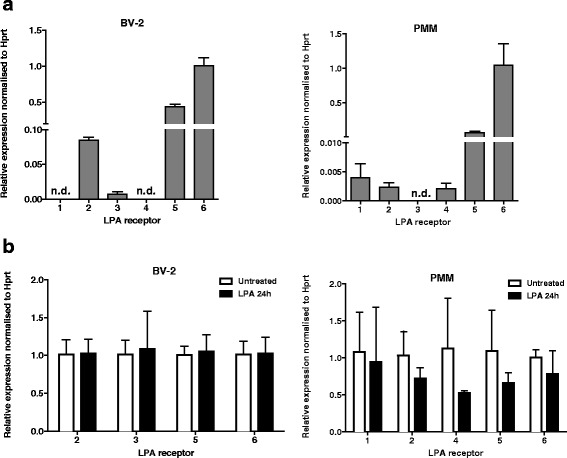
LPA receptor expression in BV-2 cells and primary murine microglia (PMM). **a** Gene expression was monitored by qPCR and normalized to the housekeeping gene Hprt in BV-2 and PMM. Values are expressed as mean + SD (*n* = 6–9). LPA6 expression was arbitrarily set to 1. *n.d.* not detected. **b** LPA receptor expression after treatment with 1 μM LPA for 24 h. Results are expressed as mean + SD from three independent experiments

LPA receptor expression in BV-2 and PMM was analyzed by qPCR. LPA2, LPA3, LPA5, and LPA6 were detected in BV-2 cells. LPA1 and 4 were undetectable (n.d.), whereas LPA3 was detected at very low copy numbers. In PMM LPA1, 2, 4, 5 and 6 were detected. LPA3 was undetectable (Fig. 1a). LPA6 expression was arbitrarily set to 1. To test whether LPA receptor expression changes upon cell activation, BV-2 and primary cells were incubated in the presence of LPA (1 μM). After 24 h, LPA receptor expression was analyzed by qPCR. These experiments (Fig. 1b) revealed that LPA is without effects on LPA receptor mRNA expression in BV-2 cells. LPA tended to decrease LPAR mRNA in PMM; however, these effects were statistically not significantly different from untreated cells.

**Fig. 2:**
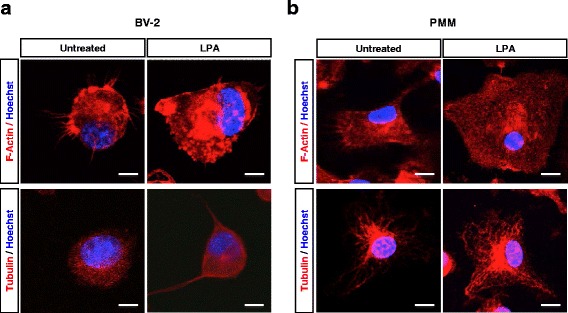
LPA alters microglia morphology. Staining for F-actin and β-tubulin in **a** BV-2 cells and **b** primary murine microglia (PMM**).** Cells on chamber slides were serum starved (o/n) and incubated in the absence or presence of LPA (1 μM, 24 h). After washing and incubation with primary and secondary antibodies, cells were analyzed by confocal microscopy. Nuclei were stained with DAPI. Representative images reveal rearrangement of the cytoskeleton following LPA treatment. *Scale bar* = 20 μm. Results from one representative experiment (out of two) are shown

Microglia are able to change their morphology in response to extracellular cues. Immunofluorescence studies revealed that LPA induced morphological changes of BV-2 (Fig. 2a) and PMM (Fig. 2b). F-actin staining (Fig. 2, upper panels) indicated that untreated cells were unipolar with one or more processes. After LPA treatment, the cells increased their surface area and acquired a flat morphology with more condensed actin labeling. Also the tubulin stains (Fig. 2, lower panels) indicated an increase in cell area in response to LPA treatment. Tubulin labeling changed from a diffuse pattern in untreated BV-2 cells to a more dense network in response to LPA. In primary cells, tubulin staining was also more condensed and occupied a larger area after LPA treatment.

**Fig. 3:**
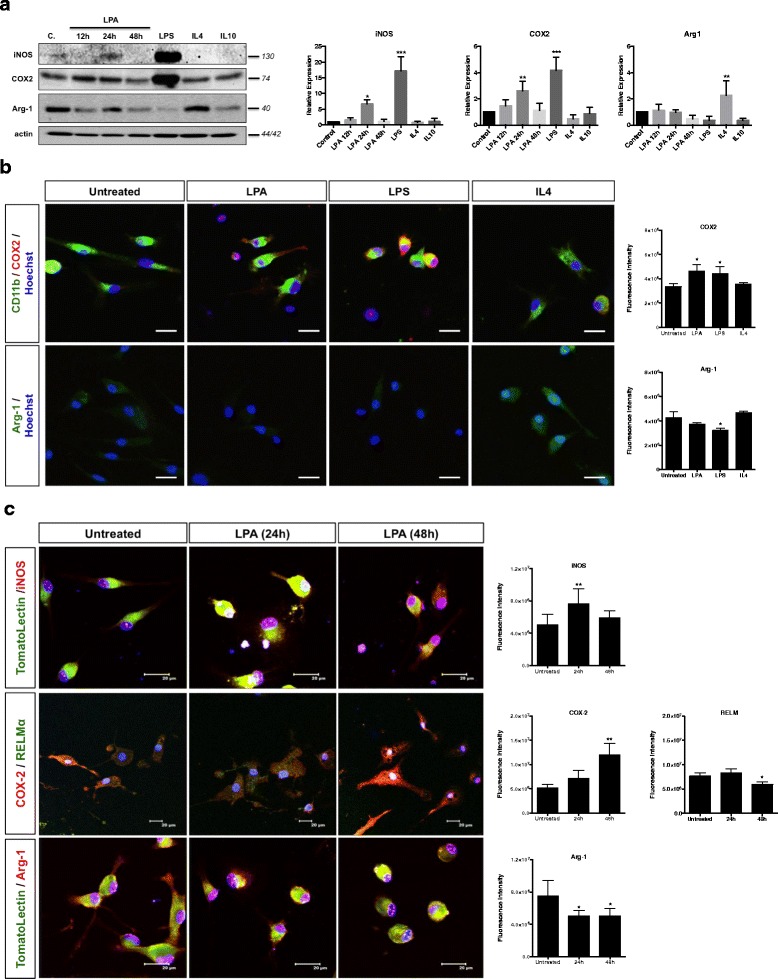
LPA promotes classical activation of BV-2 and primary microglia. **a** Serum-starved BV-2 cells were treated with BSA (0.1 %; ‘*c*.’), LPA (1 μM), LPS (20 ng/ml), IL-4 (40 ng/ml), or IL-10 (40 ng/ml)], and cellular protein lysates were analysed by Western blotting. LPS, IL-4, and IL-10 were used to polarize cells to an M1- or M2-like phenotype, respectively. One representative plot for each protein and the densitometric analysis (mean + SD; normalized to actin) from four independent experiments is presented. Control = 0.1 % BSA. **b** Confocal immunofluorescence microscopy of PMM in the absence or presence of LPA (1 μM, 24 h). LPS (20 ng/ml) and IL-4 (40 ng/ml) were used to induce an M1- or M2-like phenotype, respectively. Cells were stained for CD11b (microglia marker) and COX-2 or Arg-1. Nuclei were counterstained with Hoechst. *Scale bars* = 20 μm. Results from one representative experiment (out of two) are shown. **c** PMM cultured on chamber slides were incubated in the absence or presence of LPA (1 μM) for 24 and 48 h. Cells were stained for specific inflammatory markers and nuclei were counterstained with Hoechst. The fluorescence intensity for each marker was quantitated with ImageJ. At least 50 cells out of 3 different areas per chamber were measured in two independent experiments. The results are presented as mean + SD (*p < 0.05, **p < 0.01, ***p < 0.001; one-way ANOVA with Bonferroni correction)

Next, we evaluated the effects of LPA on BV-2 polarization by Western blot analyses to get an indication about time-dependent changes of M1/M2 marker expression. These analyses revealed that LPA increased the expression of iNOS and COX-2 (maximum induction at 24 h), but did not affect the M2 marker Arg-1 (Fig. 3a). As expected, stimulation of BV-2 cells with LPS (20 ng/ml) significantly increased iNOS and COX-2 levels. In contrast, IL-4 (a polarization signal towards M2) induced Arg-1 without affecting iNOS and COX-2 levels. IL-10 was without effect on iNOS and COX-2 levels but slightly decreased Arg-1 levels. The bar graphs in the right panel represent densitometric evaluation of the indicated protein bands.

Primary microglia were treated with LPA, LPS, or IL-4 and analyzed by confocal laser scanning microscopy for COX-2 and Arg-1 immunoreactivity. LPA treatment (1 μM, 24 h) led to increased COX-2 immunofluorescence that was mainly detected in cellular processes (Fig. 3b, upper panel). LPS induced a more rounded cell shape of PMM, and COX-2 staining was observed along the cell periphery. COX-2 expression was unaffected by IL-4. LPA (and LPS) treatment decreased Arg-1 staining, which was increased in response to IL-4 (Fig. 3b, lower panel). The bar graphs in the right panel show fluorescence intensities of micrographs displayed at the left.

In time-dependent studies, LPA increased iNOS fluorescence intensity in PMM (Fig. 3c) by 1.6-fold (24 h). Using a double M1/M2 staining approach (COX-2 and RELMα), we detected 2.3-fold increased fluorescence intensity for COX-2 (at 48 h), while RELMα fluorescence was reduced by 22 % (48 h). Arg-1 expression was also reduced in response to LPA treatment (by 35 %). The right panel shows fluorescence intensities.

**Fig. 4:**
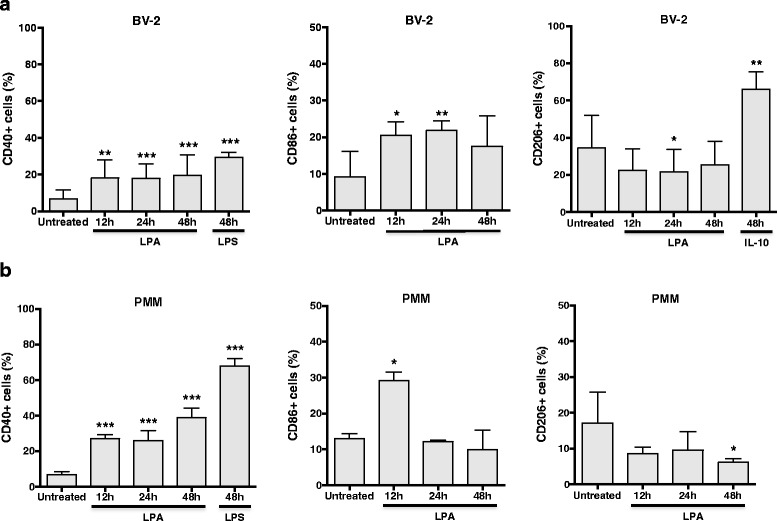
LPA induces a pro-inflammatory microglia phenotype. **a** Serum-starved BV-2 cells (o/n) were treated with 1 μM LPA for the indicated time points. LPS (20 ng/ml) and IL-10 (40 ng/ml) were used as positive controls to induce M1- and M2-like phenotypes, respectively. Cells were stained with PE anti-CD40, APC anti-CD86 or PE anti-CD206 and analyzed using a FACSCalibur flow cytometer. Results (six separate experiments in triplicate) are expressed as mean + SD (*p < 0.05, **p < 0.01, *** *p* < 0.001; one-way ANOVA with Bonferroni correction). **b** Serum-starved PMM (o/n) were cultivated in the presence of LPA (1 μM) for the indicted times. LPS was used as a positive control. Cells were stained with PE-conjugated anti-CD40, APC-conjugated anti-CD86, or PE-conjugated anti-CD206 antibodies and analyzed using a Guava easyCyte 8 Millipore Flow Cytometer. Results from three individual preparations (measurements performed in duplicate) are shown as mean values + SD (*p < 0.05, *** *p* < 0.001 compared to untreated cells; one-way ANOVA with Bonferroni correction)

We then analyzed surface marker expression by flow cytometry in LPA-treated BV-2 cells and PMM. LPA increased the percentage of CD40+ BV-2 cells from 7 to 20 % (Fig. 4a). LPS (20 ng/ml) increased the CD40+ population from 7 to 29 %. CD86 expression was increased approx. twofold in response to LPA (12 and 24 h), while CD206+ cells were reduced from 35 to 22 % (24 h). Primary microglia (Fig. 4b) showed a comparable though more pronounced response: LPA increased the percentage of CD40+ cells from 6 to 39 % (48 h), while LPS increased CD40+ cells to 67 %. The CD86+ cell population increased from 13 to 29 % (12 h) and then decreased to ≈12 % (48 h). The CD206+ population was reduced from 17 to 6 % (48 h).

**Fig. 5:**
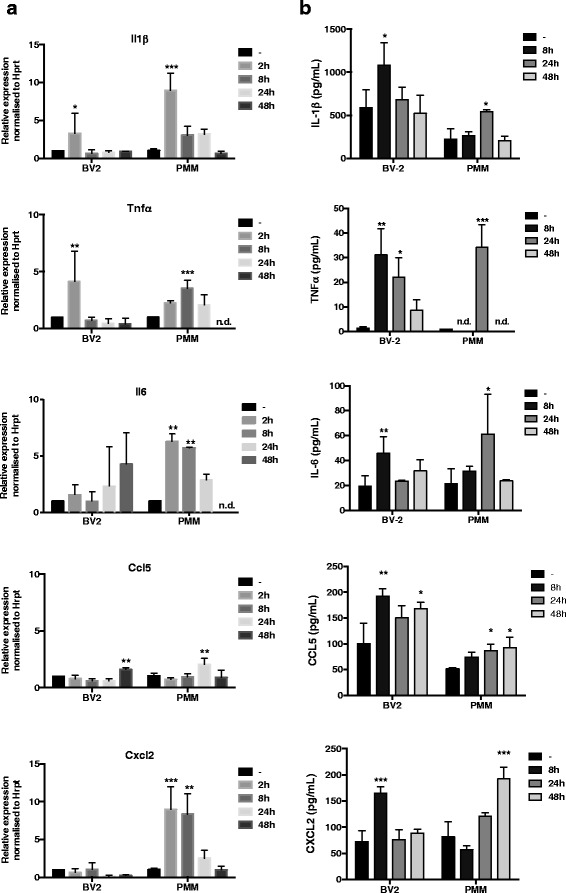
LPA induces expression and secretion of pro-inflammatory cytokines and chemokines. **a** BV-2 and PMM were cultured on 24-well plates and serum-starved (untreated) or incubated in the presence of 1 μM LPA for the indicated times. mRNA expression of different inflammatory cytokines and chemokines was monitored by qPCR and normalized to Hprt. Data are shown as mean + SD from three independent experiments performed in triplicate. Expression profiles were determined using the 2^-ddCt^ method. **b** Serum-starved microglia cells were treated with LPA (1 μM) for the indicated times. Murine ELISA kits were used to quantitate the concentrations of IL-1β, TNFα, IL-6, CCL5 (RANTES), and CXCL2 (MIP-2) in the cellular supernatants. Results shown represent mean + SD from two independent experiments performed in triplicate. Data are expressed as mean values + SD (*p < 0.05; **p < 0.01; ***p < 0.001; one-way ANOVA with Bonferroni correction)

Next, we determined the effect of LPA treatment on gene expression and secretion of selected pro-inflammatory cytokines and chemokines that are associated with an M1-like microglia phenotype [47]. qPCR analyses revealed that LPA increased transcription of Il1β, Tnfα, Il6, Ccl5, but not Cxcl2 in BV-2 cells (Fig. 5a, left panel). In PMM, LPA induced time-dependent transcription of Il1β, Tnfα, Il-6, Ccl5, and Cxcl2.

In addition, we quantitated cytokine/chemokine concentrations in the cellular supernatants by ELISA. In both cell types, LPA augmented secretion of IL-1β, TNFα, IL-6, CCL5, and CXCL2 (Fig. 5b). In BV-2 cells, analytes were maximally induced at 2 or 8 h post activation. In PMM, maximal concentrations were observed between 24 (IL-1β, TNFα, IL-6, CCL5) and 48 h (CXCL2).

**Fig. 6:**
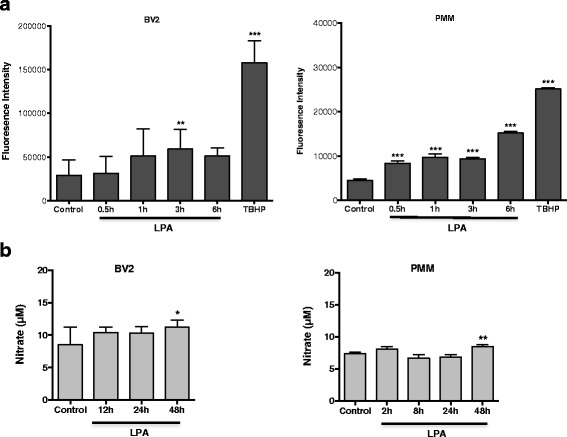
LPA increases NO and ROS production in BV-2 and primary murine microglia. **a** The cellular redox status was determined using carboxy-H_2_DCFDA. Serum-starved BV-2 cells and PMM were incubated with carboxy-H_2_DCFDA and treated with LPA (1 μM), and the fluorescence intensity was quantitated. *tert*-Butyl hydroperoxide (TBHP) was used to induce intracellular ROS formation. Results (four independent experiments performed in triplicate) are expressed as mean values + SD. (**p ≤ 0.01; ***p < 0.001; one-way ANOVA with Bonferroni correction). **b** Serum-starved cells were treated with LPA (1 μM) for the indicated time periods. The production of NO was determined by measuring nitrate concentrations. Data (three independent experiments performed in triplicate) are presented as mean values + SD. (*p < 0.05; **p < 0.01; one-way ANOVA with Bonferroni correction)

To analyze the intracellular redox status, we measured DCF fluorescence. These experiments revealed a twofold increase of fluorescence 3 h post LPA addition in BV-2 cells (Fig. 6a, left panel). *tert*-Butyl hydroperoxide (tBHP; an inducer of intracellular ROS formation) was used as positive control (50 μM, 6 h). The DCF response was more pronounced in PMM and time-dependently increased (3.3-fold). In these cells, tBHP increased DCF fluorescence by 5.7-fold (Fig. 6a, right panel). Nitrate concentrations (surrogate markers for NO production via iNOS) in LPA-treated BV-2 and PMM increased by 1.3- and 1.1-fold, respectively (Fig. 6b).

**Fig. 7:**
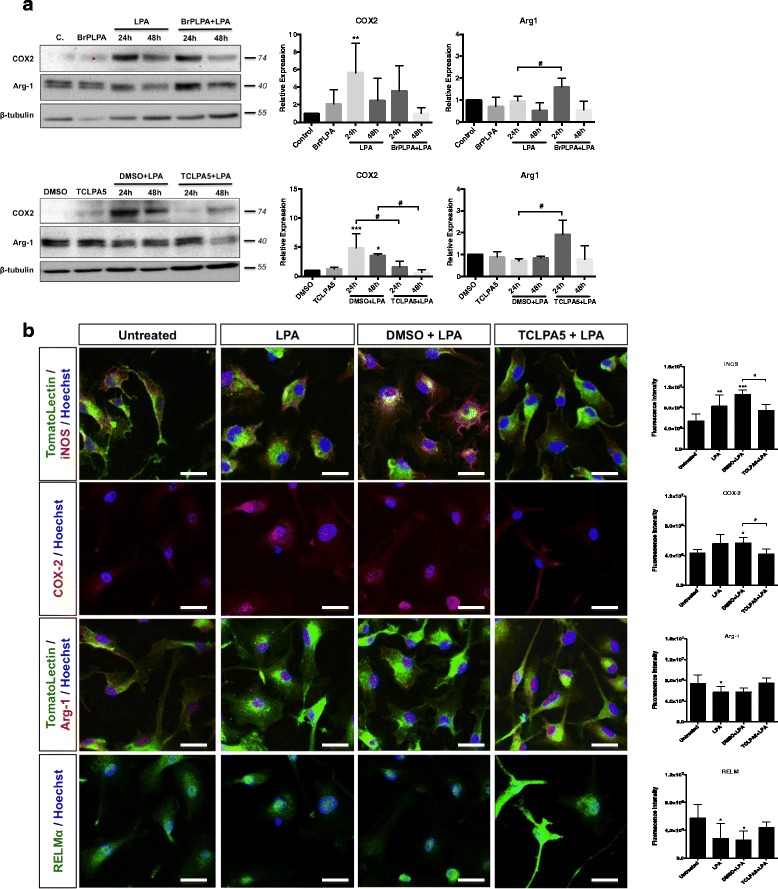
Inhibition of LPA5 suppresses the LPA-induced pro-inflammatory phenotype in BV-2 and primary murine microglia. **a** Serum-starved BV-2 cells were treated with LPA in the absence or presence of BrP-LPA (5 μM; upper panel) or TCLPA5 (5 μM; lower panel) added 2 h prior to LPA addition. COX-2 and Arg-1 response was monitored using Western blotting. One representative plot for each protein and the densitometric analysis (mean + SD) from four independent experiments is presented. (**p < 0.01; ***p < 0.001; ^#^ *p* < 0.05, inhibitor compared to LPA-treated cells; one-way ANOVA with Bonferroni correction). **b** PMM were incubated in the presence of vehicle (DMSO; 'untreated'), LPA (1 μM), vehicle (DMSO) plus LPA (1 µM), or TCLPA5 (5 μM in DMSO; added 2 h prior to LPA addition) plus LPA (1 µM) for 24 h. Cells were stained for iNOS, COX-2, Arg-1, or RELMα and visualized using confocal microscopy. Fluorescence intensity was quantitated with ImageJ. At least 50 cells out of 3 different areas per chamber were measured. Results (three independent experiments) are presented as mean + SD (*p < 0.05; ***p < 0.001; ^#^ *p* < 0.05 inhibitor compared to LPA-treated cells; one-way ANOVA with Bonferroni correction)

To get a more detailed picture which member(s) of the LPA receptor family is/are responsible for signal transmission in BV-2 and primary microglia, we used the pan LPA receptor inhibitor BrP-LPA and the LPA5 inhibitor TCLPA5. TCLPA5 was chosen since LPA5 was identified as a member of the microglia sensome [48]. To the best of our knowledge, no LPA6 inhibitor is currently commercially available. In BV-2 cells, BrP-LPA reduced LPA-induced COX2 expression (statistically not significant) and increased Arg-1 signals (Fig. 7a, upper panel). COX-2 activation by LPA was reduced by TCLPA5 at both time points analyzed (Fig. 7a; lower panel**)**. During these experiments, LPA activation was also performed in the presence of DMSO to account for potential inadvertent effects mediated by the vehicle. Bar graphs at the right show densitometry of immunoreactive bands.

To confirm the involvement of LPA5 in PMM, we performed immunofluorescence studies (Fig. 7b). These experiments revealed the expected induction of iNOS and COX-2 in response to LPA, while the M2 markers Arg-1 and RELMα were decreased. In response to TCLPA5, iNOS and COX-2 expression was significantly reduced with Arg-1 and RELMα being unaffected. The right panel shows fluorescence intensities of the corresponding micrographs.

**Fig. 8:**
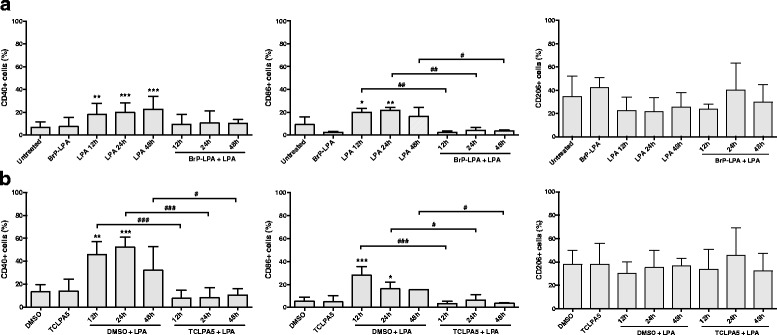
LPA receptor antagonists attenuate M1 surface marker expression in BV-2 cells. Serum-starved (o/n) cells were cultivated in the presence of vehicle, LPA (1 μM), or LPA plus **a** BrP-LPA (5 μM) and **b** TCLPA5 (5 μM) for the indicated times. Inhibitors were added 2 h prior LPA addition. Cells were stained with PE-conjugated anti-CD40, APC-conjugated anti-CD86, or PE-conjugated anti-CD206 antibodies and analyzed using a Guava easyCyte 8 Millipore flow cytometer. Results from four individual experiments in triplicate are shown as mean values + SD. (*p < 0.05; **p < 0.01; ***p < 0.001 compared to untreated or DMSO-treated cells; ^#^ *p* < 0.05; ^##^ *p* < 0.01; ^###^ *p* < 0.001 inhibitor compared to LPA-treated cells; one-way ANOVA with Bonferroni correction)

Surface marker expression analyses in LPA-stimulated BV-2 in the absence or presence of BrP-LPA and TCLPA5 is shown in Fig. 8. CD40 expression was lower in the presence of BrP-LPA (though not significant) whereas CD86 levels were significantly decreased at all time points analyzed (Fig. 8a). The percentage of CD206+ cells was unaffected by BrP-LPA. The presence of TCLPA5 during LPA activation significantly reduced the CD40 and CD86 positive cell populations to baseline levels at all time points analyzed while CD206 was unaffected (Fig. 8b).

**Fig. 9:**
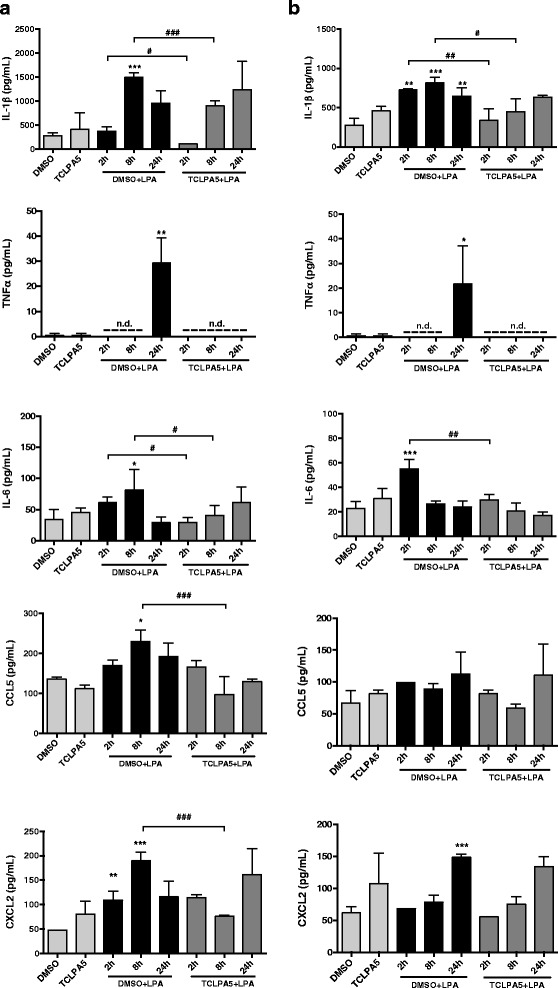
TCLPA5 inhibits the secretion of pro-inflammatory cytokines and chemokines. **a** BV-2 and **b** PMM were cultured on 24-well plates and serum-starved o/n. The supernatants were collected after incubation with vehicle, 1 μM LPA, or LPA plus TCLPA5 (5 μM) for the indicated times. The concentrations of IL-1β, TNFα, IL-6, CCL5 (RANTES), and CXCL2 (MIP-2) were quantitated by ELISA. Results shown represent mean + SD from two independent experiments performed in triplicate (*p < 0.05; **p < 0.01; ***p < 0.001 compared to vehicle control; ^#^ *p* < 0.05, ^##^ *p* < 0.01; ^###^ *p* < 0.001 TCLPA5 compared to LPA treated cells; one-way ANOVA with Bonferroni correction)

Cytokine secretion in response to LPA in BV-2 cells (Fig. 9a and PMM (Fig. 9b showed a general tendency to be reduced when TCLPA5 was present (Fig. 9). Secretion of all cyto-/chemokines was significantly reduced in BV-2 at one (TNFα, CCL5, and CXCL2) or two (IL-1β, IL-6) time points (Fig. 9a). In contrast, TCLPA5 inhibited secretion of IL-1β, TNFα, and IL-6 but was without effect on CCL5 and CXCL2 in PMM (Fig. 9b).

**Fig. 10:**
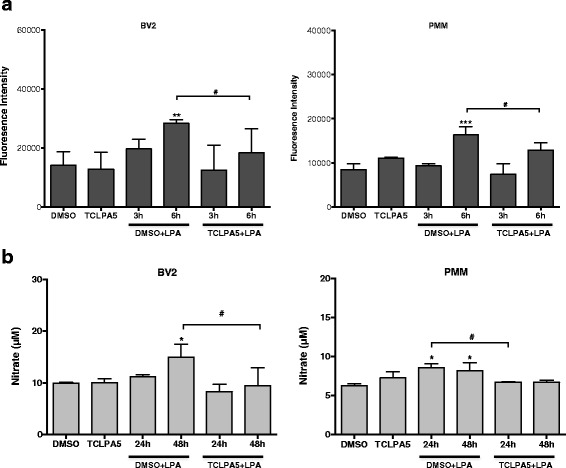
TCLPA5 suppresses ROS and NO production. **a** The intracellular ROS levels generated by BV-2 and PMM were determined using carboxy-H_2_DCFDA. Serum-starved BV-2 and PMM were incubated with carboxy-H_2_DCFDA, treated with vehicle control, LPA (1 μM), or LPA plus TCLPA5 (5 μM) for the indicated time periods, and the fluorescence intensity was evaluated. Results (three independent experiments performed in triplicate) are presented as mean values + SD. (**p < 0.01; ***p < 0.001 compared to vehicle; ^#^ *p* < 0.05 compared to LPA treated cells; one-way ANOVA with Bonferroni correction). **b** Serum-starved BV-2 and PMM were incubated with vehicle (DMSO), LPA (1 μM), or LPA plus TCLPA5 (5 μM) for the indicated times, and the production of NO was determined by measuring the total nitrate concentration in the supernatants. Data (three independent experiments performed in triplicate) are presented as mean values + SD. (*p < 0.05; compared to untreated cells; ^#^ *p* < 0.05 compared to LPA treated cells; one-way ANOVA with Bonferroni correction)

Finally, we determined the effects of TCLPA5 on ROS and NO formation in both cell types. TCLPA5 significantly reduced LPA-mediated DCF fluorescence at 6 h in BV-2 and primary microglia (Fig. 10a). Nitrate concentrations were reduced at 48 (BV-2) and 24 h (PMM) by TCLPA5 (Fig. 10b).

## Discussion

In the present study, we provide first evidence that LPA polarizes BV-2 and PMM towards an M1-like phenotype. Of note, the responses in BV-2 and primary microglia were qualitatively comparable, indicating that BV-2 cells represent a suitable prescreening model. Our findings might have bearings in neurological disease settings where LPA levels are increased, e.g., in spinal cord injury, traumatic brain injury, or multiple sclerosis [21]. LPA receptor profiling in the present study confirmed low expression of the classical LPA1-3 receptors [28] (and LPA4) but revealed high expression of LPA5 and LPA6. All of the LPA receptors are expressed in the developing brain and expression levels vary with developmental age [21]. LPA-mediated processes regulate proliferation, microtubule-dependent interkinetic nuclear migration, neurite retraction, cell survival, morphological changes, and cell migration [21]. Thus, in physiological conditions, LPA-mediated signaling contributes to normal development and function of the CNS.

However, in response to injury, LPA levels can rise significantly in the brain and CSF [8, 17, 19, 49, 50]. LPA levels are elevated in human (0.05 controls vs. 0.27 μM post injury) and mouse (0.8 and 2 μM, prior vs. post injury) CSF in response to traumatic brain injury [51]. In this context, it is of importance that exogenous LPA can fuel endogenous LPA production via an LPA3-dependent pathway [52]. Overshooting LPA signaling has been linked to the development of fetal hydrocephalus in embryonic mice, a pathophysiological process that is ameliorated in LPA1/LPA2 double knockout animals [50]. LPA signaling is also involved in nerve injury-triggered pain responses [53], where LPA1 [54] and LPA5 [55] contribute via independent mechanisms. Findings that LPA5 is activated during nerve injury (but not under basal conditions) are consistent with the fact that LPA levels rise significantly in response to spinal cord injury [8, 19]. In the contused spinal cord, parenchymal LPA concentrations increase from 75 to 725 pmol/mg protein (naïve vs. 3 days post injury) and contribute to secondary injury manifested as demyelination [19]. Demyelination in the injured spinal cord was (at least in part) ascribed to LPA-activated microglia [19]. Lysophosphatidylcholine injected intrathecally is converted to LPA via ATX-mediated pathways, and an LPA3-dependent feed-forward loop induces further endogenous synthesis of LPA [52]. It was suggested that within this setting, microglia activation is responsible for de novo LPA synthesis and concomitant development of neuropathic pain [56]. Thus, findings of the present study that LPA induces an M1-like microglial phenotype are relevant to pathophysiology in the injured/diseased CNS.

Here we show that LPA induces the expression of an M1 signature in BV-2 and primary microglia. In line with a previous study [47], LPS induced iNOS, COX-2, CD40, and CD86 expression. LPA treatment resulted in the upregulation of the M1 markers iNOS, COX-2, CD40, and CD86 and downregulation of the M2 markers CD206 (MRC1), Arg-1, and Relmα in BV-2 and primary microglia. The transcriptional programs that drive an LPA-mediated M1 signature are currently under investigation. Classical M1 marker expression was accompanied by increased cytokine/chemokine mRNA and protein levels as well as ROS and NO production in BV-2 and PMM. In PMM, LPS increased iNOS and COX-2 expression as well as IL-6 release [47] in a similar manner as observed here for LPA-treated BV-2 cells and PMM. Also, the temporal expression profiles are in agreement to what was reported for M1 marker expression in LPS-treated PMM: Gene expression of iNOS and COX-2 was significantly elevated between 4 and 72 h, and protein levels of IL-6 were significantly elevated over baseline up to 72 h [47]. The 2.5-fold increase in IL-6 secretion of primary microglia in response to LPA observed here is in a comparable range reported for LPA-stimulated fibroblast-like synoviocytes [57], which play an active role in synovial inflammation and damage via ATX/LPA-mediated pathways. iNOS is not expressed in the healthy brain, but expression is induced in response to inflammatory mediators like LPS or cytokines. Increased ROS and NO concentrations make it reasonable to assume that, in response to iNOS upregulation, excess NO reacts with NADPH oxidase-derived O_2_^−^. This reaction results in the formation of the highly neurotoxic peroxynitrite (ONOO^−^) in BV-2 microglia [58]. It is important to note that DCF (used during the present study to detect alterations in cellular redox balance) is not a species-specific probe but is, in addition to H_2_O_2_, also oxidized by hypochlorous acid (generated via the myeloperoxidase/H_2_O_2_/chloride system), other peroxidases, and ONOO^−^ [59]. ONOO^−^ was shown to induce mitochondrial dysfunction in neurons [60] to damage oligodendrocytes [61] and to compromise blood-brain barrier function [62]. Although our results suggest that LPA stimulation leads to ROS and NO production and is potentially neurotoxic, Awada and colleagues [63] have shown that overexpression of ATX in BV-2 microglia protects cells against H_2_O_2_-induced cell damage and oxidative stress. The same group [64] demonstrated ATX-mediated downregulation of cytokine production (mRNA and protein) in LPS-stimulated BV-2 cells. These seemingly contradictory results to the present study might be simply due to different incubation/culture conditions: In the ATX overexpression model [63], BV-2 cells are continuously exposed to LPA concentrations that are approx. fourfold elevated over the vector controls in contrast to the single addition used in the present study. Although we show that exposure to a single LPA bolus does not change LPA receptor expression in BV-2 cells and PMM, the situation might be different in ATX-overexpressing microglia. In terms of downregulated cytokine production in ATX-overexpressing BV-2 cells, Awada et al. [64] used LPS-stimulated cells while we studied effects of LPA on BV-2 and PMM that were exposed only to LPA (in the absence of a co-agonist). This is reminiscent of what was reported for peritoneal macrophages [65]: In that study, LPA induced IL-6 but not TNFα secretion in unstimulated macrophages while in LPS-stimulated cells, LPA downregulated TNFα but not IL-6 production. Thus, it appears that LPA-mediated effects depend on the cellular preactivation experience resulting in altered responsiveness upon rechallenge probably related to the intrinsic immune memory of microglia [66].

Time-dependent gene/protein expression of cytokines/chemokines have profiles specific for an M1 or M2 microglia phenotype [47]. We observed upregulated expression of IL-1β, IL-6, Tnfα, and the immunomodulatory chemokines CCL5 and CXCL2 in response to LPA treatment, findings reminiscent of what was reported for LPS-activated PMM [47]. In particular, increased IL-1β, IL-6, and TNFα concentrations were linked to a poor prognosis in infants suffering from ischemic encephalopathy [67]. In addition, IL-1β, IL-6, TNFα, and the chemokines CCL5 and CXCL2 (which were all upregulated by LPA in primary microglia) are implicated as modulators of the neuroinflammatory response during traumatic brain injury [68] where LPA levels are increased [51].

To get first indications about LPA receptors that are responsible for signal transmission in microglia, we have performed pharmacological inhibitor studies. BrP-LPA is a pan LPA receptor/ATX inhibitor [44] while TCLPA5 is a specific antagonist of LPA5 [45]. Of note, both inhibitors suppressed M1 marker expression in BV-2 and PMM and attenuated LPA-stimulated cytokine secretion. Since TCLPA5 blunted all pro-inflammatory signals, it is conceivable that LPA5 is a major player in LPA-dependent M1 polarization of microglia. Our findings are in line with LPA5-mediated signaling cascades in immunocompetent cells including a sensome function in microglia [48]. Using a novel chemical probe acting as specific antagonist for LPA5, it was shown that this receptor induces Ca^2+^ release from LPA-stimulated BV-2 cells in response to hexadecyl-LPA [69]. In line with results of the present study, Kozian and colleagues [69] demonstrated highest expression for LPA5 in BV-2 cells (LPA6 is not mentioned in this article) and reported a sub μM IC50 (730 nM) for this novel LPA5 antagonist for Ca^2+^ release in 16:0 alkyl-LPA stimulated BV-2 cells. Currently, no published data are available whether commercially available TCLPA5 or the LPA5 antagonist described in [69] crosses the blood-brain or blood-cerebrospinal fluid barriers to foster application in CNS disease models. In human mast cells, LPA5 is essential for conveying signals leading to MIP-1β (CCL4) generation and secretion underlining the importance of this signaling route as regulator of cyto- and/or chemokine production [70].

**Fig. 11:**
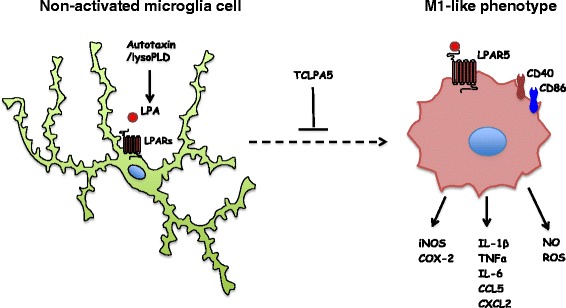
Graphical summary of findings obtained during the present study. LPA treatment induces a unique pro-inflammatory M1-like signature in BV-2 and PMM that is completely or partially reversed by pre-treatment with the LPA5 inhibitor TCLPA5. Data of the present study provide functional evidence for the role of LPA5 as member of the microglia sensome

Based on the present results (graphically summarized in Fig. 11), we hypothesize that interference with the LPA/LPA5 signaling axis might provide an opportunity to pharmacologically shift microglia polarization. However, the therapeutic usefulness of such an approach has to be carefully evaluated in in vivo studies.

## Conclusions

We conclude that our data provide functional support for a sensome function of LPA5 in BV-2 and PMM. The present in vitro study indicates that interference with the LPA signaling axis either at the level of LPA synthesis (using, e.g., ATX inhibitors) or at the level of signal transmission (LPA receptor antagonists) could offer new means to modulate the microglia polarization status.

## Abbreviations

APC: Allophycocyanin
Arg-1: Arginase 1
ATX: Autotaxin
BrP-LPA: 1-Bromo-3(S)-hydroxy-4-(palmitoyloxy)butyl-phosphonate
CXCL2: C-X-C motif chemokine 2
CCL4: C-C motif chemokine 4
CCL5: C-C motif chemokine 5
CNS: Central nervous system
COX-2: Cyclooxygenase 2
DCFDA: 2′,7′-dichlorofluorescin diacetate
DMSO: Dimethylsulfoxide
IL-1β: Interleukin-1β
IL-6: Interleukin-6
iNOS: Inducible nitric oxide synthase
LPA: Lysophosphatidic acid
LPA1-6: LPA receptors 1-6
MRC1: Macrophage mannose receptor 1
ONOO^−^: Peroxynitrite
o/n: Overnight
PE: Phycoerythrin
PMM: Primary murine microglia
qPCR: Quantitative real-time PCR
RELMa: Resistin-like alpha
ROS: Reactive oxygen species
TBHP: *tert*-Butylhydroperoxide
TCLPA5: 5-(3-Chloro-4-cyclohexylphenyl)-1-(3-methoxyphenyl)-1H-pyrazole-3-carboxylic acid
TNFα: Tumor necrosis factor α
